# OBUSight: Clinically Aligned Generative AI for Ophthalmic Ultrasound Interpretation and Diagnosis

**DOI:** 10.1002/advs.202515864

**Published:** 2026-01-08

**Authors:** Xiaocong Liu, An Shao, Bingtao Guan, Ziyao Luo, Weiyi Lai, Xiaoling Huang, Jun Liu, Jie Yan, Huimin Li, Xiangji Pan, Jiawei Wang, Zichang Su, Yih Chung Tham, Jie Yang, Haotian Lin, Juan Ye, Hongxia Xu, Jian Wu

**Affiliations:** ^1^ State Key Laboratory of Transvascular Implantation Devices of the Second Affiliated Hospital Zhejiang University School of Medicine and Transvascular Implantation Devices Research Institute Hangzhou China; ^2^ Zhejiang University, Eye Center of Second Affiliated Hospital School of Medicine, China Zhejiang Provincial Key Laboratory of Ophthalmology Zhejiang Provincial Clinical Research Center for Eye Diseases Zhejiang Provincial Engineering Institute on Eye Diseases Hangzhou China; ^3^ Liangzhu Laboratory and WeDoctor Cloud Zhejiang Key Laboratory of Medical Imaging Artificial Intelligence Hangzhou China; ^4^ Zhongshan Ophthalmic Center, Sun Yat‐sen University, WHO Collaborating Centre for Eye Care and Vision, State Key Laboratory of Ophthalmology, Guangdong Provincial Key Laboratory of Ophthalmology and Visual Science Guangdong Provincial Clinical Research Center for Ocular Diseases Guangzhou China; ^5^ Department of Ophthalmology National University of Singapore Singapore Singapore; ^6^ Singapore Eye Research Institute Singapore National Eye Centre Singapore Singapore; ^7^ Centre for Innovation and Precision Eye Health National University of Singapore Singapore Singapore; ^8^ Division of Pharmacoepidemiology and Pharmacoeconomics Department of Medicine Brigham and Women's Hospital and Harvard Medical School Boston Massachusetts USA; ^9^ Department of Genetics and Biomedical Informatics Zhongshan School of Medicine Institute for Frontier Interdisciplinary Research in Health Sciences and Technology Sun Yat‐sen University Guangzhou China; ^10^ Hainan Eye Hospital and Key Laboratory of Ophthalmology, Zhongshan Ophthalmic Center Sun Yat‐sen University Guangzhou China

**Keywords:** disease diagnosis, generative artificial intelligence, multimodal learning, ocular B‐scan ultrasonography, report generation

## Abstract

Ocular B‐scan ultrasonography (OBU), widely used for diagnosing posterior segment ocular disorders, poses unique challenges for ophthalmologists in image interpretation. In this study, a clinically aligned generative artificial intelligence (AI) model, OBUSight, was proposed to jointly generate reports and diagnose diseases for comprehensive OBU image interpretation. OBUSight was trained and validated on a large multi‐center OBU dataset consisting of 39 654 images and 17 586 corresponding reports from 11 381 patients. By evaluating the quality of generated reports using natural language generation (NLG) metrics and clinical efficacy (CE) metrics, OBUSight outperformed eight state‐of‐the‐art models and demonstrated robust performance across multi‐center and multimorbidity validation datasets. The expert rating further indicated that OBUSight can provide clinically aligned reports without major corrections. The ancillary role of OBUSight in enhancing diagnostic efficiency was evaluated by providing ophthalmologists, residents, and ophthalmology students with its generated reports and predicted diagnoses during the diagnostic process. In both retrospective and prospective evaluations, OBUSight significantly outperformed residents and ophthalmology students (all *p* < 0.05), achieved diagnostic performance comparable to ophthalmologists, and reduced diagnostic time. In conclusion, OBUSight represents a promising AI tool for enhancing diagnostic efficiency in ophthalmic ultrasound practice, especially for less experienced clinicians.

## Graphical abstract

OBUSight, a clinically aligned generative AI model that jointly generates reports and predicts diseases through multimodal semantic alignment, was trained and validated on a large multicenter dataset. OBUSight outperformed eight state‐of‐the‐art models, provided clinically reliable reports, enhanced diagnostic efficiency, and achieved performance comparable to ophthalmologists, particularly benefiting less experienced clinicians.

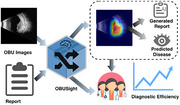

## 1 Introduction

Ocular B‐scan ultrasonography (OBU) is a non‐invasive imaging technique that uses high‐frequency sound waves to visualize the internal structures of the eye and orbit, particularly when visualization is hindered by media opacities [1]. It has been widely applied in screening and diagnosing various ophthalmic diseases, especially for posterior segment ocular disorders [2]. Compared to other ophthalmic imaging techniques, OBU presents unique challenges for ophthalmologists in image interpretation, clinical reporting, and disease diagnosis. First, OBU produces grayscale, low‐contrast images often with blurred boundaries and limited structural definition, making it difficult to distinguish anatomical features [3]. Second, OBU lacks standardized acquisition protocols, making the quality of OBU images highly dependent on the operator's experience, probe angle, and patient cooperation [4]. Third, OBU images are generated based on sound wave reflection, which depends on the acoustic impedance of tissues. Since different pathologies may share similar tissue densities, it is challenging to distinguish between diseases [5].

Artificial intelligence (AI) technologies have shown great promise in medical image interpretation, particularly with pretrained foundation models [6, 7, 8] and generative AI [9, 10] such as ChatGPT [11] and DeepSeek [12]. Studies have demonstrated that these AI technologies can effectively assist physicians in interpreting various medical images, thereby enhancing human‐in‐the‐loop clinical decision‐making [13, 14, 15]. Although AI applications in ophthalmology have been widely explored using modalities such as color fundus photography and optical coherence tomography images [6, 7, 15], research on OBU remains limited [16]. Existing OBU studies have primarily applied convolutional neural networks for posterior segment pathology detection [16, 17, 18, 19, 20, 21, 22] or automatic segmentation [4, 23], with emerging interest in automated report generation. Wang et al. presented a multimodal OBU dataset comprising images, blood flow information, and examination reports to support automated report generation [24], while Gan et al. developed a multimodal AI system for automatic OBU report generation and interactive interpretation support [25]. However, these studies typically address disease classification and report generation as isolated tasks, rather than within an integrated framework that reflects real‐world clinical decision‐making. This limitation hampers the exploration of effective human‐AI collaboration within actual clinical workflows. Moreover, most existing methods for both disease classification and report generation rely solely on image inputs.

Recently, multimodal learning has been increasingly adopted for report‐generation systems. CLIP‐based architectures [26, 27] such as EyeCLIP [28] and RetiZero [29] leveraged contrastive learning to capture shared representations across images and reports. BLIP‐based architectures such as FFA‐GPT [30] and OphthUS‐GPT [25] combined a visual encoder with a bidirectional text encoder‐decoder for cross‐modal representation learning. Other cross‐modal frameworks usually integrate a visual encoder, a textual encoder, and a fusion module [31, 32]. Nonetheless, these approaches generally overlook the expert‐curated lesion descriptions in clinical reports that are essential for clinically meaningful interpretation. This leads to insufficient semantic alignment among images, textual reports, lesion labels, and diagnostic information, ultimately restricting the model's ability to attend to clinically relevant regions. The rise of generalist generative medical AI, especially vision language models (VLMs), has expanded capabilities in medical reporting and downstream tasks, but these systems still face limitations, including susceptibility to hallucinations and biases, particularly when the clinical context is incomplete [10]. While the recently proposed EyeCLIP [28] was trained on multiple ophthalmic imaging modalities, it contains only a small amount of OBU data and has not been evaluated on OBU‐specific tasks. These limitations highlight the need for OBU‐specific, clinically aligned multimodal systems that more closely reflect real diagnostic workflows.

**FIGURE 1:**
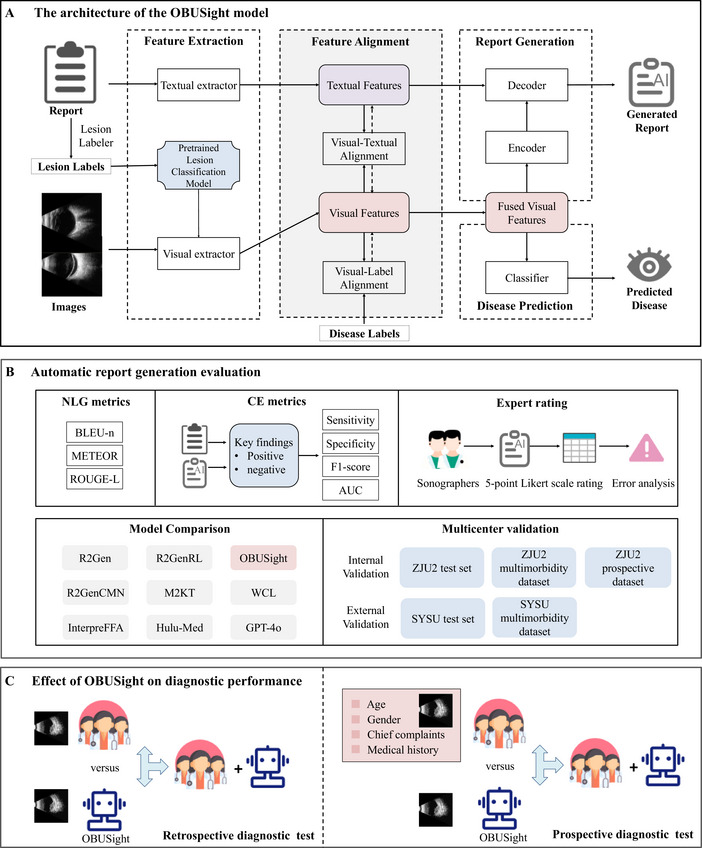
Overview of study design. (A) The architecture of the OBUSight model. The OBUSight model includes four components: feature extraction, feature alignment, report generation, and disease prediction. (B) Automatic report generation evaluation. The generated reports were evaluated by natural language generation (NLG) metrics, clinical efficacy (CE) metrics, and expert evaluation based on a 5‐point Likert scale rating. (C) Effect of OBUSight on diagnostic performance. Three groups of clinicians with varying ophthalmological expertise: two ophthalmologists, two residents and two ophthalmology students were recruited to evaluate the OBUSight's impact on diagnostic performance by comparing clinician decision‐making with and without model assistance in both retrospective and prospective evaluations. In the prospective diagnostic evaluation, clinicians made a diagnosis based on patient age, gender, chief complaints, medical history, and OBU images.

To address these limitations, we first established a large multicenter ophthalmic ultrasound dataset containing OBU images, corresponding reports, diagnostic labels, and lesion labels extracted from the reports. Second, OBUSight, a novel lesion‐guided generative AI model, was proposed to jointly generate OBU reports and assist in disease diagnosis via multimodal semantic alignment. Third, we benchmarked OBUSight's performance against real‐world OBU reports by evaluating it comprehensively on multicenter and multimorbidity datasets in terms of both literal similarity and clinical consistency. Also, we compared OBUSight to several state‐of‐the‐art medical report generation models and visual language models. Finally, by evaluating the impact of OBUSight on the clinical diagnostic process through both retrospective and prospective validation, we aim to validate OBUSight as a promising tool for enhancing diagnostic performance and efficiency, thereby supporting the broader clinical integration of generative AI technologies. The overview study design is shown in Figure 1.

## 2 Results

### 2.1 OBUSight Development Using Multicenter Datasets

The OBUSight model, an encoder–decoder architecture with feature extraction, feature alignment, report generation, and disease prediction modules, was proposed for comprehensive OBU interpretation (Figure 1A). The model was developed and validated using 26 195 OBU images and 11 836 reports from 6978 patients retrospectively collected from the Second Affiliated Hospital, Zhejiang University School of Medicine (ZJU2). Internal testing was first conducted on 6541 images and 2958 reports from 2288 patients, followed by an additional evaluation on a multimorbidity dataset comprising 3700 images and 1331 reports from 825 patients. For external validation, two independent datasets from Zhongshan Ophthalmic Center at Sun Yat‐sen University (SYSU) were used: an external test set containing 1653 images and 779 reports from 679 patients, and a multimorbidity dataset containing 1111 images and 482 reports from 446 patients. Finally, a prospective clinical validation was performed using 454 images and 200 reports from 165 patients. The detailed demographics information and OBU data of the included datasets are summarized in Table 1.

**TABLE 1:** Summary of datasets.

Items	ZJU2 dataset					SYSU dataset	
	Training set	Validation set	Test set	Multimorbidity test	Real‐world test	Test set	Multimorbidity test
Patients	4691	2287	2288	825	165	679	446
Age (SD)	47.2 (17.6)	47.8 (17.1)	47.8 (17.3)	51.3 (14.7)	51.0 (16.0)	51.0 (17.4)	51.7 (13.4)
Gender (%)							
Male	2170 (46.2)	1069 (46.7)	1096 (47.9)	446 (54.1)	95 (57.6)	412 (60.7)	270 (60.5)
Female	2521 (53.7)	1218 (53.3)	1192 (52.1)	379 (45.9)	70 (42.4)	267 (39.3)	176 (39.5)
Eye (%)							
OS	2277 (48.5)	1081 (47.3)	1141 (49.9)	387 (46.9)	91 (55.2)	379 (55.8)	245 (45.1)
OD	2414 (51.4)	1206 (52.7)	1147 (50.1)	438 (53.1)	74 (44.8)	300 (44.2)	201 (54.9)
Images	19648	6547	6541	3700	454	1653	1111
Reports	8876	2960	2958	1331	200	779	482
Report length (SD)	51.6 (13.7)	51.6 (13.4)	51.5 (13.9)	70.8 (17.8)	52.0 (16.7)	32.9 (6.4)	48.9 (16.3)
Diagnosis (%)							
CD	258 (2.9)	83 (2.8)	90 (3.0)	318 (23.9)	27 (13.5)	32 (4.1)	104 (21.5)
GT	325 (3.7)	129 (4.4)	99 (3.3)	28 (2.1)	4 (2.0)	—	—
IFB	31 (0.3)	9 (0.3)	10 (0.3)	12 (0.9)	17 (8.5)	6 (0.8)	11 (2.3)
Normal	138 (1.6)	34 (1.1)	47 (1.6)	—	7 (3.5)	—	—
OT	41 (0.5)	9 (0.3)	9 (0.3)	20 (1.5)	—	—	93 (19.3)
PSS	3962 (44.6)	1353 (45.7)	1314 (44.4)	880 (66.1)	50 (25.0)	241 (30.9)	122 (25.3)
RB	195 (2.2)	68 (2.3)	73 (2.5)	84 (6.3)	6 (3.0)	—	59 (12.2)
RD	1287 (14.5)	397 (13.4)	426 (14.4)	1117 (83.9)	32 (16.0)	200 (25.7)	345 (71.6)
SOT	1455 (16.4)	482 (16.3)	525 (17.7)	68 (5.1)	7 (3.5)	34 (4.4)	—
VH	1184 (13.3)	396 (13.4)	365 (12.3)	485 (36.4)	50 (25.0)	266 (34.1)	276 (57.3)

CD: choroidal detachment; GT: gas tamponade; IFB: intraocular foreign body; OD: oculus dexter; OS: oculus sinister; OT: orbital tumor; PSS: posterior sclera staphyloma; RB: retinal break; RD: retinal detachment; SD: standard deviation; SOT: silicone oil tamponade; VH: vitreous hemorrhage. In the multimorbidity datasets, disease labels are counted multiple times, as each case is associated with multiple conditions.

### 2.2 Automatic Report Generation Evaluation

The performance of report generation was automatically evaluated using natural language generation (NLG) metrics and clinical efficacy (CE) metrics (Figure 1B). We found that OBUSight outperformed eight state‐of‐the‐art medical report generation models and visual language models in the internal test set, achieving satisfactory performance in both NLG metrics and CE metrics (Table 2A). The reports generated by OBUSight exhibited a high degree of literal similarity to the ground truth reports, achieving a BLEU‐4 score of 0.783, a METEOR score of 0.577, and a ROUGE‐L score of 0.859. Also, OBUSight effectively identified key ophthalmic findings in OBU images, accurately describing them in generated reports with an AUC of 0.907. As detailed in Table S1, OBUSight excels in identifying the categories of “pseudo‐distention” (AUC: 0.996) and “rectangular depression of the posterior globe wall” (AUC: 0.959).

**TABLE 2:** Automatic report generation metrics in internal test set and validation performance of OBUSight across different datasets.

A										
Model	NLG Metrics						CE Metrics			
	BLEU‐1	BLEU‐2	BLEU‐3	BLEU‐4	METEOR	ROUGE‐L	Sensitivity	Specificity	F1‐score	AUC
M2KT	0.780	0.749	0.729	0.710	0.529	0.808	0.749	0.968	0.786	0.858
R2Gen	0.841	0.811	0.785	0.764	0.566	0.847	0.819	0.971	0.830	0.885
R2GenRL	0.803	0.757	0.721	0.691	0.515	0.791	0.757	0.963	0.775	0.860
R2GenCMN	0.845	0.815	0.791	0.771	0.568	0.855	0.829	0.963	0.826	0.886
WCL	0.819	0.789	0.766	0.747	0.549	0.837	0.775	0.973	0.808	0.874
InterpreFFA	0.852	0.821	0.796	0.776	0.570	0.856	0.823	0.969	0.829	0.896
Hulu‐Med	0.012	0.003	0.001	0.000	0.043	0.076	0.034	**0.992**	0.065	0.513
GPT‐4o	0.234	0.037	0.011	0.001	0.195	0.185	0.426	0.935	0.550	0.680
OBUSight	**0.867**	**0.834**	**0.806**	**0.783**	**0.577**	**0.859**	**0.850**	0.969	**0.833**	**0.907**
**B**										
Dataset	NLG Metrics						CE Metrics			
	BLEU‐1	BLEU‐2	BLEU‐3	BLEU‐4	METEOR	ROUGE‐L	Sensitivity	Specificity	F1‐score	AUC
ZJU2 multimorbidity test	0.72	0.648	0.597	0.555	0.494	0.754	0.727	0.924	0.732	0.826
ZJU2 real‐world test	0.725	0.677	0.643	0.616	0.522	0.764	0.872	0.898	0.754	0.885
SYSU test	0.331	0.238	0.183	0.133	0.287	0.406	0.798	0.912	0.76	0.855
SYSU multimorbidity test	0.307	0.197	0.139	0.086	0.196	0.306	0.662	0.877	0.649	0.769

**FIGURE 2:**
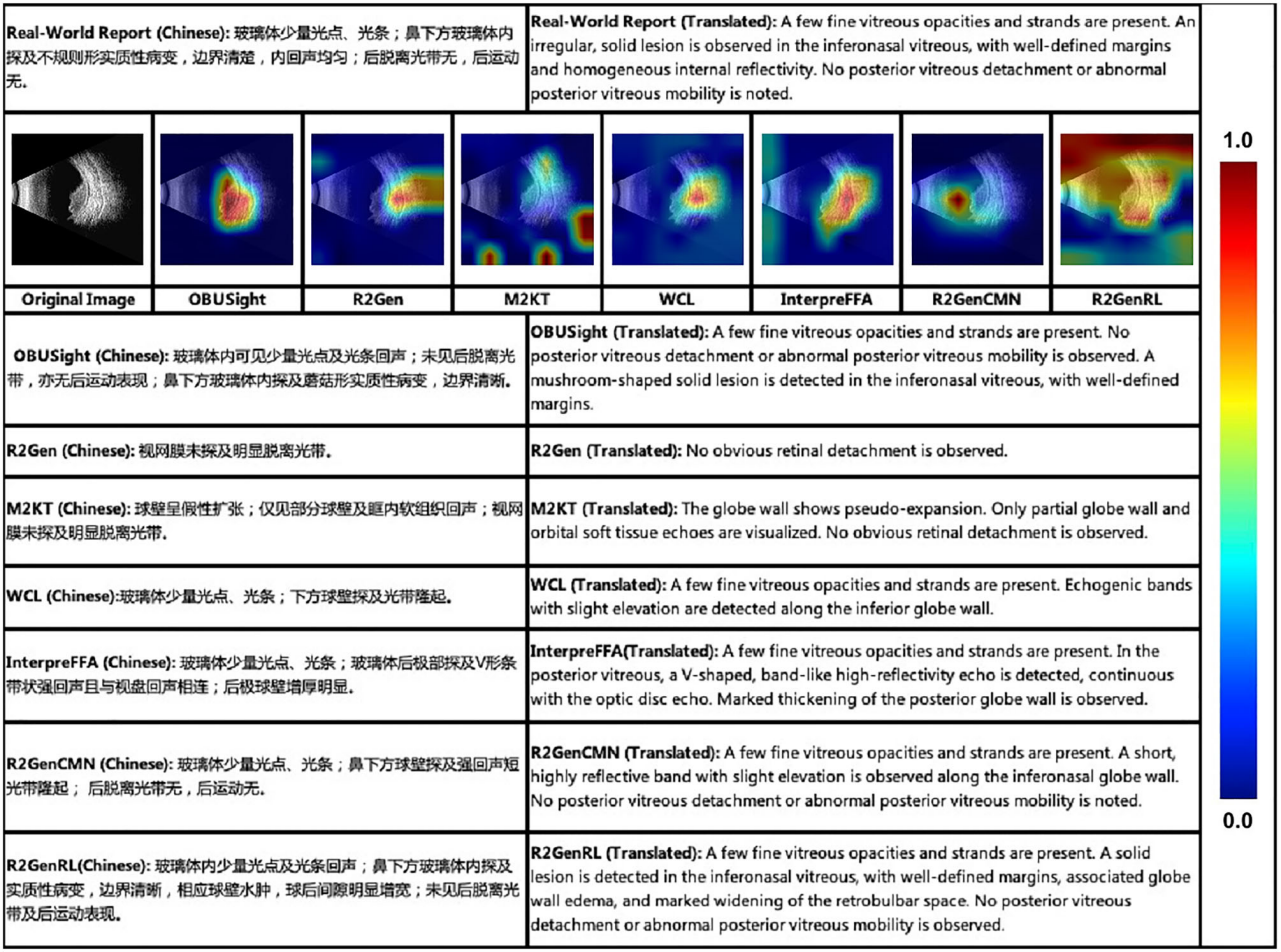
Interpretability visualization of OBUSight model in an orbital tumor case.

To further qualitatively compare different models and validate our lesion‐guided learning strategy, we visualized the image‐text attention mappings extracted from the multi‐head attentions in the final decoder layer. As illustrated in Figure 2, our model can more accurately localize lesions associated with the indicated diseases and generate high quality reports. This interpretability provides valuable insight into the OBUSight's reporting and diagnostic mechanisms, enhancing physicians’ trust and facilitating broader adoption in real‐world clinical practice.

To ensure the generalizability and clinical applicability of our model, we performed an internal validation on a multimorbidity dataset, followed by two external validations using an independent test set and an external multimorbidity dataset. In addition, an internal prospective validation was performed to further assess the model's clinical utility. As shown in Table 2B, OBUSight demonstrated comparable and satisfactory performance on both NLG and CE metrics for the internal multimorbidity dataset and the prospective dataset, demonstrating its capability to handle more complex real‐world clinical scenarios. For the two external datasets which were collected using different imaging devices and exhibited distinct reporting styles, OBUSight showed suboptimal performance on NLG metrics but achieved comparable CE performance, with an AUC of 0.855 on the external test set and 0.769 on the external multimorbidity dataset. These findings indicate that although our model has limited ability to generate reports consistent with varying external reporting styles, it can effectively identify key ophthalmic findings from OBU images acquired from different imaging devices.

### 2.3 Expert Evaluation of AI‐Generated Reports

**FIGURE 3:**
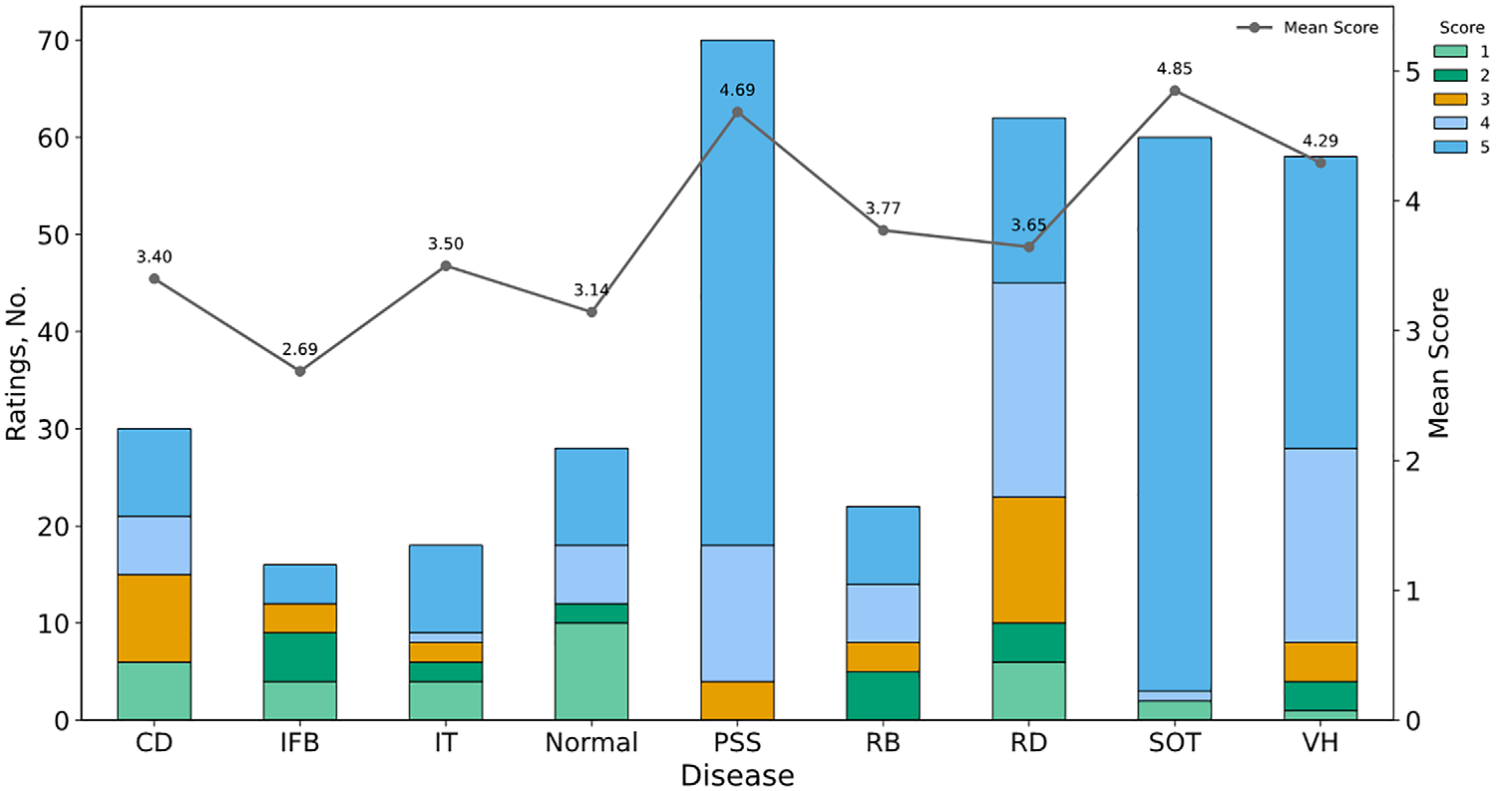
The distribution of 5‐point Likert scale ratings. CD: choroidal detachment; GT: gas tamponade; IFB: intraocular foreign body; OT: orbital tumor; PSS: posterior sclera staphyloma; RB: retinal break; RD: retinal detachment; SOT: silicone oil tamponade; VH: vitreous hemorrhage.

Two specialized sonographers were invited to rate AI‐generated reports based on the 5‐point Likert scale (Table S2), assessing the degree of agreement in the clinical consistency. The results of Cohen's weighted kappa coefficient demonstrated strong agreement between raters (Cohen's k = 0.923). The averages of expert's scores were 4.03 ± 0.07, significantly higher than neutral value of 3 (*p* < 0.001). Additionally, reports for most diseases received significantly higher ratings (all *p* < 0.001), while others showed moderate quality, especially intraocular foreign body (IFB) only with 2.69 ± 0.28 score (Figure 3).

We also found that 74.5% of AI‐generated reports (rated as 5 or 4) were accepted or with minor changes. For reports rated 3 or lower, error analysis is presented in Figure S1. Notably, 10.0% (rated 1) had to be completely discarded, mostly normal cases misclassified as posterior sclera staphyloma (PSS), likely due to the absence of the characteristic posterior fundus outpouching in some instances.

### 2.4 Diagnostic Performance in the Retrospective Validation Study

We assessed the impact of OBUSight on diagnostic performance by simulating clinician workflows with and without model assistance in the retrospective diagnostic test involving two ophthalmologists, two residents, and two ophthalmology students from ZJU2. Table 3 presents a detailed comparison of the diagnostic performance between OBUSight and human experts with or without AI assistance. OBUSight achieved an accuracy of 77.55% (95% CI: 71.50%–83.01%) and an F1‐score of 75.99% (95% CI: 69.49%–82.37%) for ten clinical diseases. Moreover, Cohen's kappa between our model and the ground truth labels (Cohen's k = 0.740) exceeded that of all clinicians. Notably, OBUSight achieved performance comparable to that of ophthalmologists and statistically outperformed residents and ophthalmology students (all *p* ≤ 0.008).

**TABLE 3:** Comparison of diagnostic performance between our model and human experts, with and without model assistance in the retrospective internal test set.

Method	Accuracy (%)	F1‐Score (%)	Kappa (%)	P‐value	Time cost per case (second)
OBUSight	77.55 (71.50‐83.01)	75.99 (69.49‐82.37)	74.04 (67.34‐80.57)	/ ^a^	
Human experts					
Student 1	53.41 (46.50–60.50)	51.78 (44.68–59.22)	46.62 (39.07–54.27)	< 0.001	34.2
Student 2	49.02 (42.50–55.50)	48.97 (42.07–56.30)	41.99 (34.76–49.32)	< 0.001	29.4
Resident 1	62.96 (56.00–69.50)	63.83 (56.88–70.50)	58.02 (50.59–65.29)	0.002	23.4
Resident 2	66.80 (59.50–73.50)	65.80 (58.34–72.68)	61.93 (53.91–69.17)	0.008	20.4
Ophthalmologist 1	70.51 (63.50–76.50)	68.80 (61.30–75.57)	66.11 (58.88–72.97)	0.120	13.8
Ophthalmologist 2	73.43 (67.50–79.00)	73.19 (66.78–78.96)	69.52 (62.82–75.90)	0.332	9.9
Human experts with model assistance					
Student 1	68.51 (62.00–74.50)	67.16 (60.44–73.34)	63.61 (56.65–70.41)	0.008	13.2
Student 2	66.89 (60.50–73.01)	65.99 (59.65–72.64)	61.96 (54.77–69.04)	0.003	9.3
Resident 1	73.58 (67.49–79.00)	73.69 (67.35–79.44)	69.73 (62.48–76.08)	0.312	13.2
Resident 2	76.49 (70.50–82.50)	75.98 (69.76–81.94)	73.05 (66.39–79.65)	0.880	12.9
Ophthalmologist 1	78.77 (72.50–84.00)	78.05 (71.72–83.88)	75.58 (68.69–81.72)	0.788	11.1
Ophthalmologist 2	80.36 (74.99–85.50)	79.78 (73.53–85.20)	77.39 (71.03–83.26)	0.497	12.0

^a^ The diagnostic performance of OBUSight was used as the reference. P‐value: McNemar test

With the assistance of OBUSight, all human experts showed statistically significant improvements in diagnostic performance (all *p* < 0.01, Figure S2). Despite an average increase of 16.20% in F1‐score, the ophthalmology students’ performance remained statistically significantly lower than that of OBUSight (all *p* ≤ 0.008). With OBUSight's assistance, residents’ performance approached that of the model, while ophthalmologists surpassed it by an average of 2.93% in F1‐score. Notably, the AI assistance led to an average diagnostic time reduction of 20.6 s per case for ophthalmology students and 8.9 s for residents. The abovementioned results indicated that OBUSight can help bridge the gap for less experienced clinicians and enhance the efficiency of expert workflows.

**FIGURE 4:**
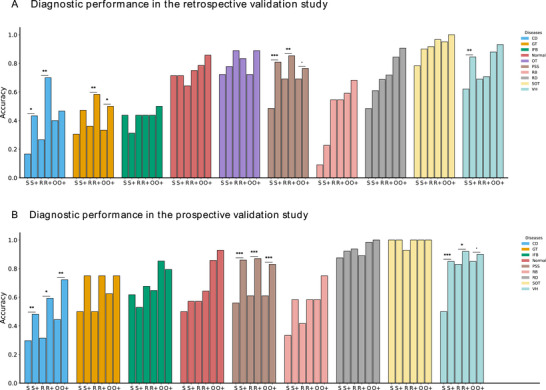
Diagnostic performance of each disease under different conditions. P‐value of per‐disease diagnostic performance: McNemar s Test. S: students; R: residents; O: ophthalmologists; +: human experts with model assistance. ^*^ *p* < 0.05, ^**^ *p* < 0.01, and ^***^ *p* < 0.001; CD: choroidal detachment; GT: gas tamponade; IFB: intraocular foreign body; OT: orbital tumor; PSS: posterior sclera staphyloma; RB: retinal break; RD: retinal detachment; SOT: silicone oil tamponade; VH: vitreous hemorrhage.

The diagnostic performance of each disease under different conditions is illustrated in Figure 4A. With the assistance of OBUSight, human experts’ diagnostic accuracy across nearly all disease types achieved varying levels of improvement. However, both OBUSight and human experts demonstrated poor diagnostic performance in identifying under‐represented disease IFB with extremely low prevalence rates of 0.35% in the training set. Additionally, on OBU images, IFB typically manifests as highly echogenic punctate or linear echoes, often with posterior acoustic shadowing. These characteristics can be easily misinterpreted as other ocular pathologies, indicating that IFB is inherently difficult to diagnose based on OBU images alone.

### 2.5 Diagnostic Performance in the Prospective Validation Study

A prospective diagnostic test was conducted at ZJU2 using a diagnostic platform to compare the diagnostic performance of the OBUSight model with that of clinicians. Unlike the retrospective validation, clinicians in the prospective study diagnosed diseases based on a comprehensive set of clinical information, including patient age, gender, chief complaints, medical history, and OBU images. As shown in Table S3, OBUSight achieved a diagnostic accuracy of 79.03% (95% CI: 73.50%–84.51%). Its agreement with ground truth labels (Cohen's k = 0.739) surpassed that of all clinician groups. OBUSight statistically outperformed residents and ophthalmology students (all *p* < 0.05) and improved diagnostic accuracy for most disease types (Figure 4B). Notably, in this prospective validation, diagnostic accuracy for IFB showed a marked improvement compared with the retrospective validation, underscoring the necessity of integrating the patient's chief complaint, current medical history, and examinations in clinical diagnosis.

## 3 Discussion

In this study, a clinically aligned generative AI model was proposed to generate medical reports from OBU images via multimodal semantic alignment and assist in disease diagnosis. OBUSight outperformed eight state‐of‐the‐art models in both NLG and CE metrics, and its generalizability and clinical applicability were validated in multicenter datasets and multimorbidity datasets. AI‐generated reports were further rated by experts, with most disease categories receiving significantly higher ratings. In both retrospective and prospective diagnostic studies, OBUSight reached the level of ophthalmologists and statistically outperformed residents and ophthalmology students. With OBUSight assistance, the diagnostic performance of human experts improved notably—residents approached OBUSight's accuracy, while ophthalmologists surpassed it, accompanied by reduced diagnostic time. Overall, this study highlights the potential of OBUSight as an AI‐assisted tool in ophthalmic ultrasound practice, enhancing diagnostic performance and efficiency.

Previous OBU studies have primarily employed classification and segmentation methods for lesion detection and disease diagnosis. More recently, pipelines combining automated report generation with large language model (LLM)‐based question answering have been proposed to enhance ophthalmic report interpretation [30, 33, 34]. However, like most report generation models, they were evaluated using standard NLG metrics rather than on factual accuracy or clinical diagnostic utility—both of which are critical for integration into real‐world clinical workflows. In this study, a comprehensive evaluation was conducted to assess AI‐generated OBU reports from both literal similarity and clinical consistency perspectives. Expert assessments further confirmed that OBUSight can provide clinically aligned reports. Additionally, retrospective and prospective diagnostic tests highlighted its educational value in facilitating ultrasound training for medical students and enhancing diagnostic efficiency for residents. For experienced ophthalmologists, OBUSight provides meaningful clinical support without compromising their diagnostic efficiency.

**FIGURE 5:**
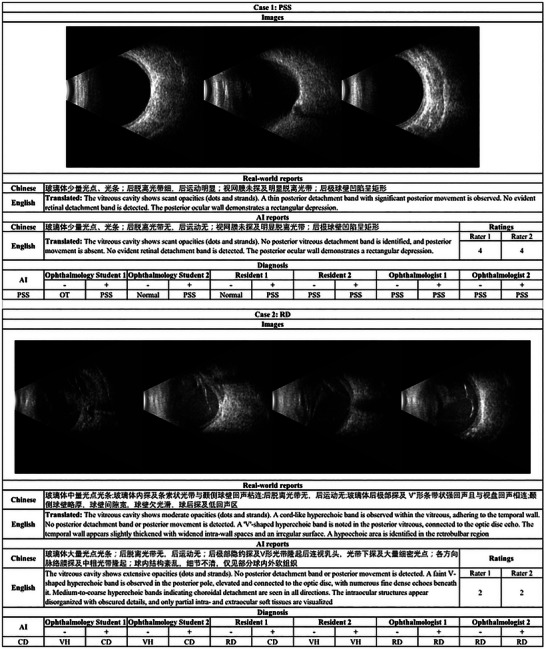
Illustration of the OBU images, reports, and results of ratings and diagnostic tests from 2 cases. ‐: human experts without model assistance; +: human experts with model assistance. PSS: posterior sclera staphyloma; OT: orbital tumor; CD: Choroidal detachment; VH: vitreous hemorrhage; RD: retinal detachment.

To specifically reveal the impact of our model, we selected 2 cases in Figure 5. In case 1, two ophthalmology students and one resident initially failed to recognize the relatively moderate outpouching of the posterior fundus and scleral bulging indicative of posterior scleral staphyloma. But after being alerted by OBUSight with the predicted diagnosis and free‐text reports, all correctly revised their diagnosis. Also, we noticed that OBUSight didn't detect the posterior vitreous detachment, and thereby two raters rates this report as 4. In case 2, the OBU images did not show the typical features of choroidal detachment (localized dome‐shaped hyperechoic elevation) but instead displayed classic signs of retinal detachment (V‐shaped hyperechoic band attached to the optic disc). OBUSight produced an incorrect diagnosis and report, which misled ophthalmology students and residents. Encouragingly, experienced ophthalmologists were not influenced by the model's error, likely due to their deep understanding of sonographic findings.

There are several limitations in our study. First, as an ancillary imaging examination, B‐scan ultrasound is clinically used to assist physicians in making a comprehensive diagnosis by integrating findings from other examinations (e.g., FFA), laboratory tests (e.g., blood test), and the patient's medical history, including chief complaints, medical history, etc. Therefore, relying solely on OBU images for disease diagnosis differs intrinsically from the real‐world clinical diagnostic process and is very challenging for all readers. Moreover, ophthalmologists can build a mental 3D map by continuously repositioning the probe while doing the OBU test, which is also different from the diagnostic task based on the static OBU images in this study. The abovementioned limitation can partially explain the relatively unsatisfied performance of ophthalmologists, residents and students participated in our diagnostic task. Future studies should incorporate additional data modalities (e.g., text information, video information) to make the diagnostic process more aligned with real‐world clinical workflows and enhance diagnostic accuracy. Second, the dataset's disease imbalance reflects real clinical settings but leads to underfitting for some rare conditions, resulting in poor diagnostic performance for low‐prevalence diseases. Third, the model has been developed and validated exclusively on Chinese‐language datasets. To improve generalizability across diverse linguistic contexts, future work may extend the text generation module through multilingual training, using both machine‐translated and native ophthalmology reports, and adopt language‐agnostic encoders that support cross‐lingual alignment. Also, future work will expand data collection to multiple institutions and incorporate domain adaptation and feature normalization strategies to further mitigate center‐specific variations. Finally, incorporating expert‐corrected diagnoses and rating feedback into the training loop represents a promising direction for further model refinement.

In conclusion, we established a large multi‐center OBU dataset and comprehensively benchmarked the proposed clinically aligned generative AI model OBUSight in OBU image interpretation. OBUSight achieved satisfactory performance, robust generalization ability, and high clinical consistency in both report generation and disease diagnosis. In diagnostic evaluations, OBUSight outperformed ophthalmology students and residents, and its assistance improved diagnostic accuracy and reduced diagnostic time for human experts. These findings underscore OBUSight's potential as an effective AI‐assisted tool to enhance clinical workflow and diagnostic efficiency in ophthalmic ultrasound practice.

## 4 Methods

### 4.1 Datasets and Preprocessing

This study was performed in accordance with the Declaration of Helsinki, and the protocol was obtained from the Ethics Committee of the Second Affiliated Hospital, Zhejiang University School of Medicine (No. Y2023‐1073) and the institutional review board of Zhongshan Ophthalmic Center at Sun Yat‐sen University (IRB‐ZOC‐SYSU, 2025KYPJ116). To develop and internally validate the OBUSight model for report generation and disease diagnosis, we collected OBU images and corresponding reports from ZJU2, including both retrospective and prospective datasets. We also included two independent external datasets from SYSU to demonstrate the generalizability of the OBUSight model. The inclusion criteria encompassed adult patients, regardless of gender or eye laterality, who presented with one or more of the following ten ocular conditions: posterior sclera staphyloma (PSS), vitreous hemorrhage (VH), retinal detachment (RD), silicone oil tamponade (SOT), gas tamponade (GT), choroidal detachment (CD), retinal break (RB), orbital tumor (OT), intraocular foreign body (IFB) and normal findings. Among these datasets, cases involving patients with multiple concurrent conditions were designated as the multimorbidity dataset to assess the model's generalizability and robustness in complex clinical scenarios. The remaining cases, retrospectively collected from ZJU2, were split into training, validation, and test sets in a 3:1:1 ratio.

The OBU images from ZJU2 were acquired using devices Compact Touch (Quantel Medical) and MD2300S (MEDA Co., Ltd.), while the OBU images from SYSU were obtained using CineScan (Quantel Medical) and Aviso (Quantel Medical). Cases with poor image quality or incomplete reports were excluded, as confirmed by experienced sonographers with over 5 years of clinical practice. To remove irrelevant peripheral information from OBU images and ensure consistency, all images were cropped into square shapes. Subsequently, all images were uniformly resized to 224 × 224 pixels to facilitate subsequent model training. For patients who underwent OBU examinations in both eyes, the raw OBU cases were separated into distinct cases for the left and right eyes. For the OBU reports written in Chinese, we randomly selected samples across all disease categories and prompted ChatGPT to generate a specialized vocabulary list, which was then reviewed by experts to ensure precise tokenization for the report generation tasks.

### 4.2 Model Development

The proposed OBUSight model takes one or multiple OBU images from a case along with the corresponding clinical report as input, and outputs both a generated report and a predicted disease label. The network architecture was based on Transformer [35], consisting of four components: feature extraction, feature alignment, report generation, and disease prediction. (Figure 1A). First, the feature extraction module includes a pretrained lesion classification model, a textual extractor, and a visual extractor. The pretrained lesion classification model was used to extract lesion‐guided features from OBU images. By referencing the CheXpert labeling software [36], we constructed a lesion labeler to extract the multi‐hot labels from the OBU reports that indicate the presence of key pathological findings. Notably, the presence of positive and negative expressions was also considered. The textual extractor was used to extract embeddings from the ground truth reports, and the visual extractor was used to extract image embeddings from the input OBU images. Second, the feature alignment consists of two parts: visual‐textual alignment and visual‐label alignment. The visual‐textual alignment module aligns paired OBU image features and corresponding report features by minimizing the triplet margin loss. The visual‐label alignment module enforces consistency among visual features associated with the same disease label using the cross‐entropy loss. If OBU cases contained multiple images, the aligned visual features were fused through an adaptive weighted attention mechanism, in which attention weights were computed via a softmax layer. Third, the fused visual features were subsequently passed through the Transformer encoder and combined with the aligned textual features in the decoder to generate the OBU report. Fourth, the fused visual features were input to a diagnostic classifier to predict the disease label.

In implementation, we first pretrained a Resnet‐101 model [37] for OBU lesion classification using self‐supervised multi‐label annotations generated by the OBU labeler, achieving an F1‐score of 0.892. This pretrained model was then used as the visual feature extractor. For text, we employed a pretrained Chinese BERT model [38]. For the encoder and decoder in Transformer, each consists of three layers and eight attention heads with 512 hidden units. To enhance model robustness and reduce overfitting, we applied data augmentation to OBU images, including random rotation, cropping, and color jittering. We also adopted a class‐balanced weighted sampling strategy to mitigate the issue of class imbalance. The model was trained using the PyTorch framework on an Nvidia GeForce RTX 3090 GPU. The detailed training settings and model hyperparameters were listed at Table S4.

To evaluate the performance of the OBUSight model, we compared it with five state‐of‐the‐art radiology report generation models, including R2Gen [39], R2GenRL [40], WCL [41], M2KT [32], and R2GenCMN [24, 42]. We also compared against InterpreFFA [43], an ophthalmology‐specific model for FFA report generation; Hulu‐Med [44], a generalist medical vision‐language model trained on 14 medical imaging modalities; and GPT‐4o, a state‐of‐the‐art generalist vision‐language model capable of open‐ended medical reasoning and report generation. The prompts given to Hulu‐Med and GPT‐4o, along with OBU images: ″Based on the ophthalmic B‐scan ultrasound images, please describe the presence of abnormalities or pathological findings to generate the corresponding imaging report, and then provide the most likely ophthalmic diagnosis. If the most likely diagnosis is normal, the report should explicitly state “No obvious abnormalities detected.”

### 4.3 Automatic Report Generation Evaluation

We evaluated the report generation performance using natural language generation (NLG) metrics and clinical efficacy (CE) metrics (Figure 1B). The NLG metrics are widely used to assess the literal similarity and coherence of generated reports, including BLEU [45], METEOR [46], and ROUGE‐L [47], However, as recent studies have noted, these NLG metrics insufficiently capture factual correctness, limiting their clinical applicability [10, 43, 48]. Therefore, we proposed clinical efficacy metrics as quantitative measures that assess the AI's ability to identify critical imaging findings and describe them appropriately in the generated reports. Specifically, two specialized sonographers were invited to build a keyword list of key entities (pathological signs or critical imaging findings) which may occurred in the real‐world reports based on their knowledge and expertise. Then we used a rule‐based algorithm to make a binary presence/absence classification to present whether these findings existed in the images according to the reports (because reports may include claims like “Adhesion” cannot be observed). Therefore, lesion labels can be extracted from both real‐world and AI‐generated reports. By comparing the consistency of these labels between the two sources, CE metrics can be calculated to quantify the AI's ability in capturing the key pathological signs (critical imaging findings), which serves as a measure of clinical correctness. Table S5 detailed the experts’ predefined key pathological findings of OBU images.

### 4.4 Expert Evaluation of AI‐Generated Reports

The clinical accuracy of the AI‐generated reports was further evaluated by experts using a 5‐point Likert scale, assessing the degree of agreement on the identification of pathological findings. The detailed scoring criteria for the Likert scale in this study were provided in Table S2. Specifically, two specialized sonographers with 10 years of clinical experience served as independent raters. They had access to ground truth reports alongside the corresponding OBU images and diagnoses. Each rater independently evaluated all 200 cases in a randomized order, ensuring that each case received 2 ratings. Additionally, for cases rated 3 or lower, raters completed a multiple‐choice form to facilitate error analysis.

### 4.5 Effect of OBUSight on Diagnostic Performance

We evaluated the effect of OBUSight on diagnostic performance by simulating the clinician workflows with or without model assistance (Figure 1C). For the retrospective diagnostic test, we first recruited three groups of participants as independent and blinded readers with varying levels of ophthalmological expertise: two ophthalmologists (each with > 5 years of clinical experience), two residents (2 years of clinical experience), and two second‐year ophthalmology students from ZJU2. These participants were not involved in the data collection process and were unaware of the study hypothesis. Second, all participants received the same 200 OBU cases (stratified randomly selected from the test set) and diagnosed these cases simply by referring to the corresponding OBU images on a diagnostic platform that mimics the picture archiving and communication system used in the real‐world clinics. Once a reader finished a case and pressed the “next” button on the tool, the diagnostic time of this case was recorded. Third, after a 2 week washout period, all participants re‐evaluated the same 200 cases (presented in a different randomized order to mitigate the impact of cases appearing in a fixed order) with model assistance, which includes the AI‐generated reports and predicted disease labels. The diagnostic time of each case was also recorded. For the prospective diagnostic test, we provided not only the OBU images but also patient age, gender, chief complaints, and medical histories to all participants, with a total of 200 cases. The rest of the procedures were consistent with those of the retrospective diagnostic test. Diagnostic performance and time cost across expertise levels were then compared between the two settings.

### 4.6 Statistical Analysis

We evaluated the overall diagnostic performance under different experimental conditions. Confidence intervals (CI) for diagnostic metrics were estimated using the bootstrap method with 1000 iterations. The diagnostic performance of OBUSight and human experts with varying experience levels, with and without AI assistance, was statistically compared using the McNemar test for paired samples. Additionally, subgroup analyses were conducted to assess diagnostic performance across disease categories. The McNemar test was further applied to examine disease‐specific diagnostic performance by AI assistance among human experts with varying levels of experience. For 5‐point Likert scale ratings, specialized sonographers independently rated the AI‐generated reports, and the inter‐rater agreement was assessed using Cohen's weighted kappa coefficient. The mean Likert scores across all reports and disease subgroups were compared against the neutral value of 3 using the Wilcoxon signed‐rank test. In this study, p‐values less than 0.05 were considered statistically significant. All statistical analyses were conducted using Python software (version 3.8.3).

## Author Contributions

X.L. and A.S. conceived the study. Z.L. and W. Lai collected the data. B.G. reviewed the related work of AI in OBU. X.L. conducted the data preprocessing, experiments, and statistical analysis. X.L. and A.S. designed the methods for model evaluation. A.S., X.H., J.L., J.Y., H.L., X.P., J.W., and Z.S. conducted the human expert evaluation. X.L. and A.S. conducted the results analysis and wrote the first draft of the manuscript. H.L., J.Y., and Y.C.T. commented on the drafts of the manuscript. J.W., H.X., and J.Y. are corresponding authors; they provided clinical guidance and administrative, technical, and material support. X.L. and A.S. contributed equally in this study. All authors revised the manuscript and approved the submitted version.

## Funding

This research was partially supported by National Natural Science Foundation of China under Grants Nos. 82202984, T2541004, Zhejiang Key R&D Program of China under Grant Nos. 2024SSYS0026, LDT23F02023F02, Zhejiang Key Laboratory of Medical Imaging Artificial Intelligence, and the Transvascular Implantation Devices Research Institute (TIDRI) under Grant No. KY052025003.

## Conflicts of Interest

The authors declare no conflict of interest.

## Supporting information




**Supporting File**: advs73678‐sup‐0001‐SuppMat.docx.

## Data Availability

The data that support the findings of this study are available on request from the corresponding author. The data are not publicly available due to privacy or ethical restrictions.
