# Supplementary material for: OBUSight: Clinically Aligned Generative AI for Ophthalmic Ultrasound Interpretation and Diagnosis
Source: Adv Sci (Weinh). 2026 Jan 8;13(16):e15864. doi: 10.1002/advs.202515864 (PMC13042833; doi:10.1002/advs.202515864)
Supplement: Supplementary file 1 — Supporting File: advs73678‐sup‐0001‐SuppMat.docx. [file ADVS-13-e15864-s001.docx]

Supporting Information

**OBUSight: Clinically Aligned Generative AI for Ophthalmic Ultrasound Interpretation and Diagnosis**

*Xiaocong Liu†, An Shao†, Bingtao Guan, Ziyao Luo, Weiyi Lai, Xiaoling Huang, Jun Liu, Jie Yan, Huimin Li, Xiangji Pan, Jiawei Wang, Zichang Su, Yih Chung Tham, Jie Yang, Haotian Lin, Juan Ye*, Hongxia Xu*, Jian Wu**

[Table S1. The CE metrics of our model in retrospective internal test set. 2](#_Toc217400109)

[Table S2. Standard of the 5-point Likert scale. 3](#_Toc217400110)

[Table S3. Comparison of diagnostic performance between our model and human experts, with /without model assistance in a prospective diagnostic study. 4](#_Toc217400111)

[Table S4. The detailed training settings and model hyperparameters of OBUSight. 5](#_Toc217400112)

[Table S5. The predefined key findings and their distribution in retrospective internal single-disease dataset. 6](#_Toc217400113)

[Figure S1. Error distribution of reports rated 3 or lower in retrospective internal test set. 7](#_Toc217400114)

[Figure S2. Diagnostic performance of human experts with/without AI assistance in retrospective internal test set. 8](#_Toc217400115)

# Table S1. The CE metrics of our model in retrospective internal test set.

| **Key findings** | **Sensitivity** | **Specificity** | **F1-score** | **AUC** | **Count** |
| --- | --- | --- | --- | --- | --- |
| Adhesion | 0.667 | 0.948 | 0.678 | 0.807 | 435 |
| Calcification | 0.750 | 1.000 | 0.857 | 0.875 | 4 |
| Dense vitreous opacities (dots and strands) | 0.500 | 0.987 | 0.537 | 0.744 | 102 |
| Elevated hyperechoic band | 0.760 | 0.960 | 0.780 | 0.863 | 528 |
| Gas reflex | 0.687 | 0.994 | 0.739 | 0.841 | 99 |
| Mild vitreous opacities (dots and strands) | 0.906 | 0.836 | 0.880 | 0.871 | 1528 |
| Moderate vitreous opacities (dots and strands) | 0.651 | 0.909 | 0.669 | 0.780 | 699 |
| Posterior vitreous detachment | 0.915 | 0.756 | 0.811 | 0.836 | 1231 |
| Pseudodistention | 0.994 | 0.998 | 0.992 | 0.996 | 525 |
| Rectangular depression of the posterior globe wall | 0.952 | 0.966 | 0.955 | 0.959 | 1310 |
| Solid mass | 0.714 | 0.999 | 0.667 | 0.857 | 7 |
| Thickened ocular wall | 0.188 | 0.957 | 0.154 | 0.860 | 16 |
| Traction | 0.398 | 0.991 | 0.465 | 0.694 | 83 |

# Table S2. Standard of the 5-point Likert scale.

| **Score** | **Description** | **Definition** |
| --- | --- | --- |
| 5 | Agree, no change necessary | Correct identification and assessment of critical findings and mostly correct identification and assessment of non-critical findings. |
| 4 | Agree, minor changes necessary | Correct identification of critical findings and one of the following errors: 1. assessment of critical findings; 2. identification of non-critical findings; 3. massive omission of non-critical findings. |
| 3 | Agree with critical findings identification, certain changes necessary | Correct identification of critical findings and two of the following errors: 1. assessment of critical findings; 2. identification of non-critical findings; 3. massive omission of non-critical findings. |
| 2 | Disagree with critical findings identification, certain changes necessary | Incorrect identification of critical findings or three of the following errors: 1. assessment of critical findings; 2. identification of non-critical findings; 3. massive omission of non-critical findings. |
| 1 | Disagree with majority of report, new report required | Incorrect or unrelated identification and assessment of findings. |

The 5-point Likert scale used in this study was based on identification and assessment of critical and non-critical findings. A critical finding was defined to be one that can lead to a relatively specific diagnosis and change the physician’s clinical management if reported incorrectly (e.g., rectangular depression of the posterior globe wall in PSS reports). Non-critical findings referred to other conditions. Identification of findings was referred to the type of findings/lesions. Assessment of findings is the description of findings/lesions including severity, amount, shape, etc. Notably, the errors in the absolute location terms (e.g., nasal, temporal, etc.) and the sentences which describe dynamic changes (e.g., the posterior movements are observed) were not considered in the ratings because the OBU image modality loses both the absolute directional information and dynamic functional information. In real-world reports, moderate omissions of non-critical findings and assessment of findings commonly exists, therefore these omissions didn’t affect the scores.

# Table S3. Comparison of diagnostic performance between our model and human experts, with /without model assistance in a prospective diagnostic study.

^a)^/ The diagnostic performance of OBUSight was used as the reference. P-value: McNemar test

| **Model** | **ACC (%)** | **F1-Score (%)** | **Kappa** | **P** | **Time cost per image (second)** |
| --- | --- | --- | --- | --- | --- |
| **Our model** | 79.03 (73.50–84.51) | 65.67 (57.14–73.58) | 73.85 (66.96–80.64) | / ^a)^ |  |
| **Human expert** |  |  |  |  |  |
| Student 1 | 60.89 (54.00–67.50) | 56.43 (49.79–62.75) | 54.49 (47.01–61.69) | <0.001 | 31.5 |
| Student 2 | 53.65 (47.00–60.50) | 51.06 (42.81–59.33) | 45.59 (38.28–53.24) | <0.001 | 24 |
| Resident 1 | 68.86 (62.50–75.00) | 61.23 (52.57–70.19) | 62.58 (55.13–69.42) | 0.029 | 13.8 |
| Resident 2 | 67.72 (61.00–74.50) | 63.99 (53.70–71.77) | 61.21 (53.63–68.86) | 0.014 | 13.5 |
| Ophthalmologist 1 | 73.90 (67.50–79.00) | 70.23 (59.96–78.17) | 68.95 (61.81–75.08) | 0.336 | 11.4 |
| Ophthalmologist 2 | 75.96 (70.00–82.50) | 76.23 (67.50–82.87) | 71.35 (64.36–78.83) | 0.635 | 9 |
| **Human expert with AI** |  |  |  |  |  |
| Student 1 | 75.38 (69.00–81.00) | 69.22 (60.21–76.57) | 70.02 (62.31–76.58) | 0.324 | 20.1 |
| Student 2 | 75.41 (69.50–82.00) | 68.58 (59.76–76.13) | 69.80 (63.13–77.44) | 0.230 | 16.5 |
| Resident 1 | 80.94 (75.50–86.01) | 73.84 (64.76–81.38) | 76.68 (70.25–83.06) | 0.608 | 9 |
| Resident 2 | 81.49 (75.99–86.50) | 76.21 (67.27–83.47) | 77.39 (70.61–83.49) | 0.522 | 9 |
| Ophthalmologist 1 | 85.52 (81.00–90.01) | 81.72 (72.36–88.62) | 82.42 (76.88–88.05) | 0.072 | 12 |
| Ophthalmologist 2 | 87.35 (82.50–91.50) | 86.04 (78.37–91.00) | 84.72 (79.30–89.76) | 0.016 | 9 |

# Table S4. The detailed training settings and model hyperparameters of OBUSight.

| **Category** | **Parameter** | **Value** |
| --- | --- | --- |
| Data settings | Maximum sequence length of the reports | 70 |
|  | The cut off frequency for the words. | 3 |
|  | Number of labels | 10 |
| Model settings for Transformer | Dimension | 512 |
|  | Number of heads | 8 |
|  | Number of layers | 3 |
|  | Dropout rate | 0.1 |
| Training configuration | Batch size | 16 |
|  | Epochs | 30 |
|  | Monitor metric | BLEU-4 |
|  | Early stop | 10 |
|  | Label smoothing | 0.1 |
|  | Optimizer | Adam |
|  | Learning rate (visual extractor) | 0.00001 |
|  | Learning rate for the remaining parameters | 0.0001 |
|  | Learning rate scheduler | StepLR |
|  | Step size of the learning rate scheduler | 10 |
|  | Gamma of the learning rate scheduler | 0.8 |

# Table S5. The predefined key findings and their distribution in retrospective internal single-disease dataset.

| **Findings** | **Positive** | **Negative** |
| --- | --- | --- |
| Adhesion | 2211 (15.0%) | 12583 (85.1%) |
| Calcification | 17 (0.1%) | 14777 (99.9%) |
| Dense vitreous opacities | 558 (3.8%) | 14236 (96.2%) |
| Elevated hyperechoic band | 2650 (17.9%) | 12144 (82.1%) |
| Gas reflex | 554 (3.7%) | 14240 (96.3%) |
| Mild vitreous opacities | 7758 (52.4%) | 7036 (47.6%) |
| Mild vitreous opacities | 3501 (23.7%) | 11293 (76.3%) |
| Posterior vitreous detachment | 6166 (41.7%) | 8628 (58.3%) |
| Pseudodistention | 2462 (16.6%) | 12332 (83.4%) |
| Rectangular depression of the posterior globe wall | 6617 (44.7%) | 8177 (55.3%) |
| Solid mass | 49 (0.3%) | 14745 (99.7%) |
| Thickened ocular wall | 117 (0.8%) | 14677 (99.2%) |
| Traction | 418 (2.8%) | 14376 (97.2%) |

# Figure S1. Error distribution of reports rated 3 or lower in retrospective internal test set.

CFI: critical findings identification; CFA: critical findings assessment, NFI: non-critical findings identification; NFA: non-critical findings assessment; NFO: non-critical findings omission

# Figure S2. Diagnostic performance of human experts with/without AI assistance in retrospective internal test set.

*p < 0.05, **p < 0.01, and ***p < 0.001
